# HIV in semen: Still more to be learned

**DOI:** 10.1186/1742-6405-2-11

**Published:** 2005-12-03

**Authors:** Pietro L Vernazza

**Affiliations:** 1 Division of Infectious Diseases and Hospital Epidemiology, Deparmtent of Medicine, Cantonal Hospital, St. Gallen Switzerland

In 1983, during the earliest days of AIDS research, Deborah Anderson and her colleagues in Boston, Massachusetts hypothesized that AIDS was transmitted by virally-infected "Trojan horse leukocytes" in semen [1]. This prediction has been supported by numerous studies over the past two decades, although many questions remain concerning HIV infection of the male genital tract. In this issue of *AIDS Research and Therapy*, the Anderson group presents an important research tool to help address some of the critical unanswered questions in this area [2].

A series of salient studies have shaped current concepts on HIV-1 in semen. In 1984, Ho et al. described retroviral particles and infected cells in the semen of a homosexual man with AIDS [3]. Shortly thereafter Stewart et al. reported infection of four out of eight women following artificial insemination with semen from one seroconverting individual [4], leading to a mandatory semen quarantine requirement and HIV testing of semen donors in Assisted Reproduction clinics. At that time, the detection of HIV, which was still termed HTLV-III, was not routinely feasible from blood, let alone from semen. Since then, technological advances have enabled the detection and quantitation of HIV-1 RNA and proviral DNA and greatly improved our understanding of the dynamics of HIV-1 in semen and sexual transmission risks. HIV-infected white blood cells have been detected throughout the male genital tract, and in preejaculatory fluid and semen from HIV+men [5,8]; the weight of evidence suggests that sperm are not infectious [9], leading to the successful development of sperm wash procedures to reduce the risk of HIV transmission from HIV-infected men to uninfected partners through assisted reproduction techniques [10]. A combination of epidemiological and clinical research studies have determined a relationship between HIV-1 RNA viral load in semen and the risk of sexual transmission. The most important factors associated with increased HIV viral loads in semen and risk of sexual transmission are: HIV-viremia and coinfections with other sexually transmitted pathogens [11,12]. HAART dramatically suppresses HIV-1 RNA viral loads in blood and semen, but HIV-1 proviral DNA can persist in semen WBCs for months after the initiation of HAART [13]. Data from other studies showing discordantly higher levels of HIV in semen than blood in some individuals support this finding. In addition, molecular sequencing studies indicate that the male genital tract is a compartment, like the central nervous system, in which HIV-1 replication and divergent evolution can occur under the influence of local factors [14]. Several clinically important questions remain: 1) Is HIV-1 primarily sexually transmitted by infected cells, cell-free virus or both? 2) What is the origin of cell-free and cell-associated HIV-1 in semen? 3) Are men on HAART with undetectable peripheral viral loads capable of sexually transmitting drug-resistant HIV-1?

Episomal HIV-1 c-DNA, a by-product of HIV-1 infection, is currently used in clinical trials as a marker of residual viral replication and potential evolution of drug resistance mutations in viral reservoir sites in individuals on HAART [15]. Such a marker would be useful for identifying sites of HIV-1 replication in the male genital tract, and for monitoring cryptic HIV-1 infection in the genital tract of men on antiretroviral therapy. The only reported study that measured episomal HIV-1 c-DNA in blood and semen of men before and after initiation of HAART failed to detect HIV episomal 2-LTR cDNA in semen [16]. The method used to recover HIV-infected cells from semen in this study – separation of seminal WBC on Ficoll gradients – likely decreased the sensitivity of HIV episomal c-DNA detection because infected macrophages and a proportion of infected T-cells are lost through this approach. The paper by Xu et al. used a direct lysis technique optimizing recovery of DNA from HIV-infected cells in semen. Using this approach, combined with quantitative PCR and DNA sequencing, the investigators show that episomal 2-LTR cDNA is detectable in semen from a subset of men with other evidence of seminal HIV-1 infection. The marker was not detected in semen from 22 men at 1- and 6-months after peripheral viral suppression due to addition of indinavir to their ART regimen. This study is important because it provides a new tool for studying HIV infection of the male genital tract, and provides preliminary evidence that cryptic HIV-1 infection may not occur in the genital tract of men on HAART. Further studies will surely follow to confirm and extend these observations.
